# Scoping review of dementia primary prevention policies in England: do they balance reach and agency?

**DOI:** 10.1136/bmjph-2025-002631

**Published:** 2025-06-25

**Authors:** Sebastian Walsh, Jack M Birch, Richard Merrick, Lindsay Wallace, Isla Kuhn, Linda Clare, Oliver T Mytton, Louise Lafortune, Wendy J Wills, Carol E Brayne

**Affiliations:** 1 Cambridge Public Health, University of Cambridge, Cambridge, UK; 2 Newcastle University, Newcastle upon Tyne, UK; 3 Department of Community Health and Epidemiology, Dalhousie University, Halifax, Nova Scotia, Canada; 4 School of Clinical Medicine, University of Cambridge, Cambridge, UK; 5 University of Exeter, Exeter, UK; 6 University College London, London, UK; 7 Faculty of Health Sciences, McMaster University, Hamilton, Ontario, Canada; 8 University of Hertfordshire, Hatfield, UK

**Keywords:** Mental Health, Preventive Medicine, Public Health, Primary Prevention, Scoping Review

## Abstract

**Objectives:**

To ascertain the balance of dementia risk reduction policies in England, considering their reach (population-wide vs targeted at specific individuals) and agency (the level of resource required to benefit from the intervention).

**Design:**

Scoping review.

**Data sources:**

Academic databases (Medline, the Health Management Information Consortium and Overton) and the webpages of relevant national and local government agencies and associated bodies (including: the UK Government, the UK Health Security Agency, National Health Service England, National Institute for Health and Care Excellence and local governments and healthcare organisations from the East of England region) were searched.

**Eligibility criteria:**

Any written documents or service webpages from, or endorsed by, governmental organisations or arms-length bodies which describe, recommend or evaluate current or formally proposed interventions for the reduction or control of one or more modifiable risk factors for dementia were included. Policies targeted at people with existing cognitive impairment and/or dementia were excluded.

**Data extraction and synthesis:**

Data on policy description, reach and agency were extracted from identified dementia primary prevention policy documents by one author. Policies common to several organisations were grouped, and then synthesised across risk factor group and by tier of government. The numerical balance of policies (between axes of reach and agency) was compared across risk factor group and current policy/proposed status.

**Results:**

From a total of 8210 hits, 366 policy documents were included. From these, 79 distinct policies were identified, targeted at dementia (n=3), cardiovascular health (n=23), smoking and alcohol (n=17), depression and social isolation (n=12), air pollution (n=10), low formal education (n=9), hearing impairment (n=3) and traumatic brain injury (n=2). Overall, 67.1% (53/79) of current policies had population-reach, 53.2% (42/79) were considered low-agency and 39.2% (31/79) were both population-reach and low-agency.

**Conclusions:**

There is currently a policy balance between population-reach and targeted-reach, and high-agency and low-agency interventions, for dementia risk reduction in England. However, a predominance of population-reach, low-agency interventions may be required to match the scale of the challenge and improve equity.

**WHAT IS ALREADY KNOWN ON THIS TOPIC**

- Dementia risk reduction is a relatively young field, and research to date has primarily focused on interventions for high-risk individuals who require a high level of agency (personal resources and motivation) to be effective.
- Public health theory calls for greater emphasis on population-level actions and lower-agency interventions.

**WHAT THIS STUDY ADDS**

- We observed a numerically balanced distribution in England between policies with population-reach and those targeted at specific individuals, as well as between interventions requiring high-agency and low-agency to be effective.

**HOW THIS STUDY MIGHT AFFECT RESEARCH, PRACTICE OR POLICY**

- There are several population-level, low-agency interventions, including taxation, food reformulation and legislative change, which could be introduced or strengthened in order to meet the scale of dementia in England and reduce health inequalities.

## Introduction

 Dementia, a syndrome of cognitive decline affecting day-to-day functioning, is the leading cause of mortality in England and Wales^1^ and a major public health challenge. Associated societal and economic costs are high,^2^ and despite research progress, treatment options remain limited.^3 4^ However, evidence from England,^5^ and other high-income countries,^6^ suggests that age-specific rates of dementia declined around the turn of the century, indicating that dementia risk in the population can be reduced.

Addressing risk factors in order to reduce the incidence of a disorder is known as primary prevention.^7^ Risk factors can be reduced (and protective factors increased) through policies with either population-reach (broad) or individual-reach (targeted). Individual-level interventions typically offer advice, medical treatment or behaviour change interventions to people identified as being high-risk (eg, a current smoker) in order to lower their risk (eg, smoking cessation support); while population-level interventions act at a group level (eg, increasing taxation on cigarettes).^7^ Interventions can also be classified as high or low ‘agency’. Agency refers to the level of resource (including financial, cognitive and social resources) required to benefit from an intervention.^8^ Lifestyle-based behaviour change interventions are typically high-agency, while interventions that require little effort from the individual, such as acting to change societal conditions (eg, reformulation of food products) are low-agency.^79^,^11^

Most future cases of dementia typically originate from the large group who are at ‘normal’ risk, compared with the small group at ‘high-risk’, meaning that population-level interventions (which aim to lower everyone’s risk) have a greater potential to lower overall disease prevalence than interventions targeted at high-risk groups only.^7 10 12^ Further, population-reach, low-agency interventions typically act by modifying the environment to make the healthier choice the default or easier choice, meaning they have the potential to narrow health inequalities while high-agency, individual-level interventions may exacerbate them.^7^,^913 14^ Dementia prevention policymakers report that population-level interventions are harder to implement because of a lack of evidence base, because they can be politically controversial, and because they are relatively more complex to design, implement and evaluate.^15^

A recent rapid review found that dementia primary prevention research has been almost exclusively focused to date on individual-level approaches.^16^ But it is unclear if this imbalance is also true for dementia primary prevention policies. In this scoping review, we use a case study approach to identify current and proposed dementia primary prevention policies (whether or not they are badged as dementia policies explicitly, or in terms of the risk factor targeted) in England. We aim to determine the balance in the approach between population-reach and individual-reach, and high-agency and low-agency interventions. By comparing the identified policies against a recent review of effective population-level interventions for modifiable risk factors,^11^ we identify opportunities for evidence-informed dementia risk reduction policy development in England.

## Methods

The protocol for this scoping review was preregistered on Open Science Framework DOI 10.17605/OSF.IO/57N9D. This manuscript was prepared following the Preferred Reporting Items for Systematic Reviews and Meta-Analyses (PRISMA) scoping review checklist (online supplemental table 1).

### Definitions and eligibility criteria

**Figure 1:**
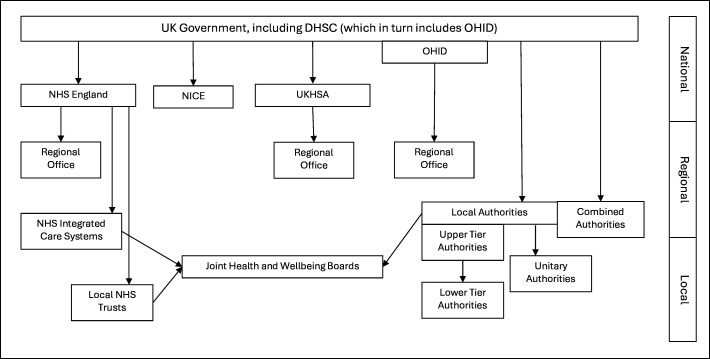
The public health system in England in 2024. DHSC, Departmet of Health and Social Care; NHS, National Health Service; NICE, National Institute for Health and Care Excellence; OHID, Office for Health Improvement and Disparities; UKHSA, UK Health Security Agency.

We included dementia primary prevention policy, strategy, (action) plan or guidance documents (hereafter collectively referred to as ‘policy documents’). We defined these as any written documents or service webpages from, or endorsed by, governmental organisations or arms-length bodies (those which are operationally independent but funded and administered by the UK Government, eg, the National Institute for Health and Care Excellence (NICE)) (figure 1), which describe, recommend or evaluate current or formally proposed interventions for the reduction or control of one or more modifiable risk factors for dementia. An explicit statement of intent to reduce dementia itself was not required.

The risk factors included were the 12 modifiable, lifecourse risk factors for dementia identified by the Lancet Commission on Dementia 2020^17^: that is, less education, hearing loss, traumatic brain injury (TBI), hypertension, alcohol consumption, obesity, smoking, depression, social isolation, physical inactivity, air pollution and diabetes.

We excluded technical documents which only provided guidance for improving existing services (eg, clinical guidelines advising on the use of one treatment over another). We excluded policies targeted at people with cognitive impairment and/or dementia, but included policies targeting people at any other stage in the lifecourse.

### Search strategy

We adapted the case study methodology used by Collins *et al* (2019).^18^ We updated their model of the English public health and preventive healthcare system (figure 1) to identify relevant organisations from which to search for policies. We used a mixed approach to policy identification, including searches of academic databases, grey literature databases and organisational websites. At the regional and local tier, it was not feasible to include every part of the country. The East of England was selected to be used as a case study because it is geographically large and diverse and includes examples of all of the organisations listed in figure 1.

For the database searches, we developed search strategies with an expert medical librarian (IK) in Medline and the Health Management Information Consortium (both via Ovid), and Overton, on the 11 June 2024, using terms related to dementia or dementia risk factors, relevant organisations, prevention and policy (see online supplemental table 2 for full search strategy). We limited results to articles published in the last 5 years, in order to identify active documents, relevant to current governance structures.

For the website searches, we separated the searches into national, regional and local levels. At the national level (England), we searched the websites of the UK Government, the UK Health Security Agency, National Health Service (NHS) England and NICE. At the regional level (East of England), we searched the websites of the 11 upper tier authorities, the tier of local government which includes a statutory public health function (county councils n=5, unitary authorities n=6); the six Health and Well-being Boards; the six NHS Integrated Care Systems; and the one Combined Authority. At the local level, we searched the websites of the 10 district authorities from one county. We tailored each search to the structure of the organisational website and the statutory responsibilities of the organisation. We used website search functions, searching for either ‘dementia’ or risk factor terms (eg, ‘smoking’) alongside filters for ‘policy’ where these existed. In addition, we manually navigated through the websites to pages where policy documents were curated. Website searches were performed between June and July 2024 (see online supplemental table 2 for full details).

### Screening process

One author (SW) conducted the searches, retrieved any potentially relevant policy/strategy documents and considered them for inclusion. A second author (JMB) screened a random 10% of documents. The two authors met to discuss and resolve conflicts; discussion with a third reviewer was not required.

### Data extraction

One author (SW) extracted data from the identified dementia primary prevention policy documents into a template which captured the details reported in online supplemental tables 3 and 4. Due to a high degree of overlap, policies targeting risk factors related to cardiovascular and metabolic health (obesity, physical inactivity, diabetes, hypertension), harmful products (alcohol and tobacco) and mental health (depression and social isolation) were each grouped, while other risk factors were considered in isolation. We categorised each policy as population-reach or individual-reach, and whether high-agency or low-agency was required to benefit (based on the definitions outlined in the Introduction section).

### Data synthesis

We grouped policies common to several organisations together, and then synthesised these across risk factor group and by tier of government. We then considered whether the numerical balance of policies (between axes of reach and agency) differed by risk factor group, or by current policy/proposed status.

### Data availability

Full screening results and extraction tables are available from the authors on request.

### Patient and public involvement in research

The University of Hertfordshire’s Public Involvement in Research group (PIRg) contributed valuable insights into the design of this study.

## Results

**Figure 2:**
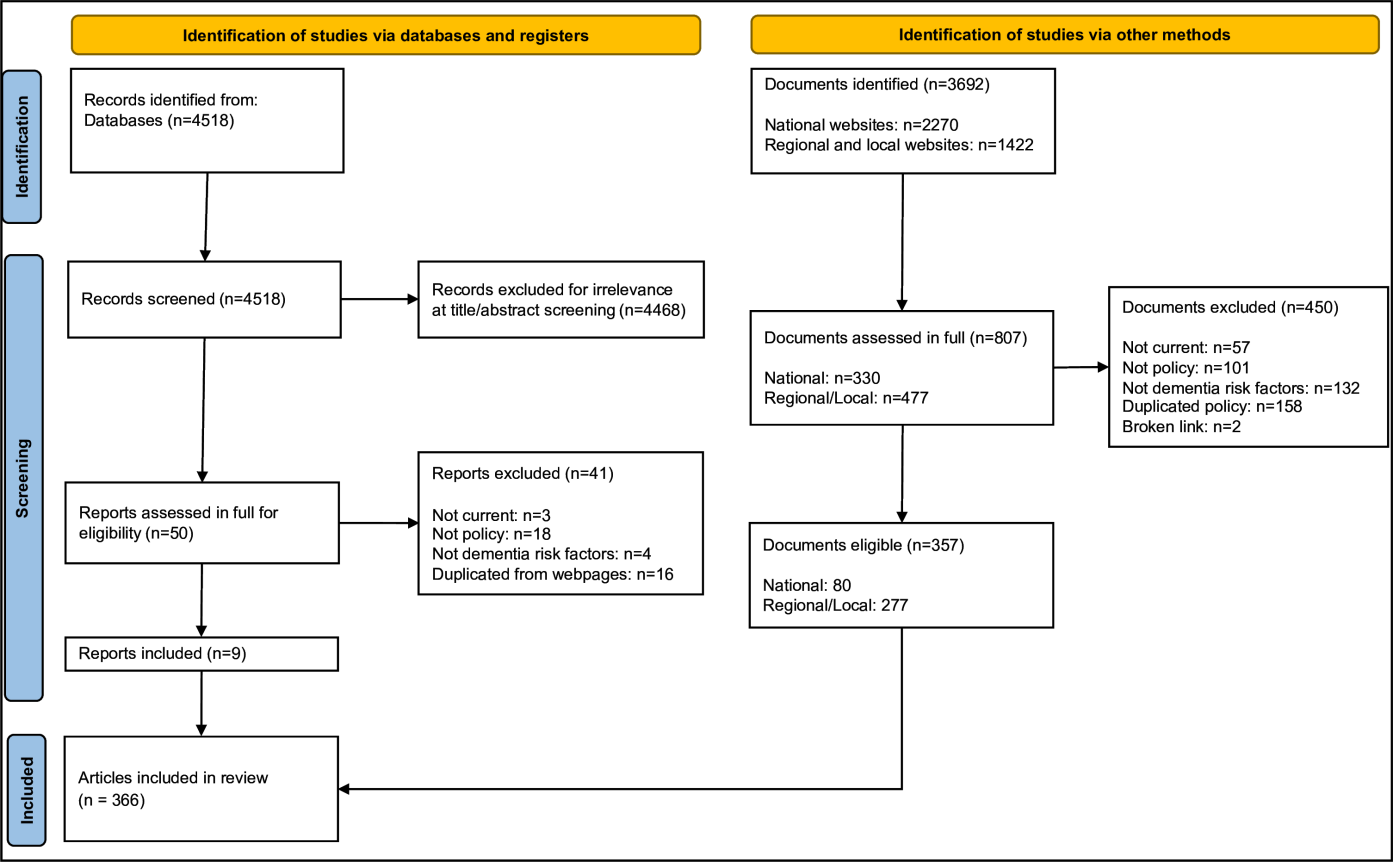
Preferred Reporting Items for Systematic Reviews and Meta-Analyses diagram showing study selection process.

We identified 4518 articles from database searches, and a further 3692 articles from organisational website searches. Of these, 857 articles were assessed in full, and 366 policy documents were included (see figure 2). There were 89 policy documents from the national tier of governance, and the remaining 277 came from regional or local agencies. From these, we extracted 79 distinct, current policies, reported in online supplemental table 3, the majority of which were common to several agencies.

### Policies by risk factor

#### Explicit dementia risk reduction policies

The only national dementia strategy for the UK was published in 2009 and focused mainly on improving early diagnosis and care services.^19^ Work on an updated strategy was rolled into a broader ‘Major Conditions Strategy’ in 2023, but this work was paused for strategic review after the 2024 change of government. So, no overarching national dementia strategy document was included.

We identified three national-level policies for dementia risk reduction (online supplemental table 3), focused on dementia awareness raising, and integrating dementia messaging into the NHS Health Check (a national cardiovascular prevention programme for 40–74 year olds) and ‘Making Every Contact Count’ (MECC) programmes (which train and encourage frontline public sector workers to provide very brief health advice in daily interactions with the public/patients). All of these had population-reach and were high-agency policies.

Many local authorities had dementia strategies. However, only two (Southend, Essex and Thurrock (SET) Dementia Strategy 2022–2026^20^ and Suffolk Dementia Strategy 2024–2029^21^) included an explicit risk reduction focus. Both had policies on raising dementia risk awareness to encourage healthy lifestyle adoption (population-reach, high-agency) (online supplemental table 3). Additionally, the SET strategy included links to various population-level strategies (such as improving cycling and walking infrastructure) being implemented by other council teams.^20^

#### Healthy weight and cardiovascular health policies

We identified 23 current policies targeting obesity, physical inactivity, hypertension and/or diabetes, across both national and local tiers of government (online supplemental table 3). Most (n=17) had population-reach, and 10 out of these were low-agency. These included food reformulation programmes, advertisement restrictions on unhealthy foods and built environment changes such as construction of active travel infrastructure. Some individual-reach policies were also considered low-agency (n=3), such as offering medical therapy to those with hypertension and diabetes, and school holiday programmes providing healthy meals and physical activity opportunities to children from low-income households. Policies requiring more agency included those with population-reach (n=7) such as NHS Health Checks, MECC and health education campaigns, and more targeted interventions (n=3) such as lifestyle interventions on referral from a clinician.

#### Alcohol and tobacco policies

We identified 17 current policies addressing alcohol and tobacco use (online supplemental table 3). Population-reach, low-agency policies (n=6) included locally enforced national bans on the sale of products to children, taxation, public place bans for smoking and advertisement/licensing restrictions. Local government policies also included targeted approaches to address social determinants of substance use, such as providing tailored housing support and job opportunities. Higher agency interventions (n=9) were similar to the cardiovascular policies described above.

#### Depression and social isolation policies

Of the 12 current policies for depression and social isolation (online supplemental table 3), only 2 were considered low-agency: both national^22^ and local^23^ strategy documents explicitly aimed to improve mental well-being through improvements to green space access and leisure services; and offering assessment services and medical therapies to those with depression through the NHS. Population-reach, high-agency interventions (n=4) included national media campaigns, funding of community-based social connector organisations (eg, community transport schemes) and school-based education programmes on mental health self-care techniques and resilience training. More targeted interventions included primary-care based ‘social prescribers’, and mental health counselling services for healthcare professionals, military personnel and based in schools.

#### TBI policies

We identified two current policies for TBI prevention (online supplemental table 3): road policies such as speed limits and traffic calming infrastructure (population-reach, low-agency), and national media campaigns, for example, ‘THINK! Bike’ (population-reach, high-agency). In addition, two of the cardiovascular health policies described above, both population-reach low-agency, were relevant to TBI prevention: building active travel infrastructure and pedestrianising roads (eg, around schools) to make them safer for active travel.

#### Hearing impairment policies

We identified three current policies addressing hearing impairment (online supplemental table 3), all of which were low-agency. Routine newborn hearing checks are available to all and trigger referral for specialist care where problems are identified, and those who present with age-related hearing loss are supported through the NHS with provision of hearing aids and/or cochlear implants, with some community services offering basic maintenance support (eg, changing batteries). National control of noise at work legislation is locally enforced to ensure reduction of noise exposure for all, and provision of ear protection where it is required.

#### Air pollution policies

All but one air pollution policy we identified (n=10) had population-reach. Mostly these were low-agency (n=7) such as legislated national targets on key pollutants, enabling local government to take enforcement action including clean air zones and anti-idling laws, and investments in/subsidies for cleaner infrastructure like electric buses. Free air pollution monitoring information and media campaigns were considered higher-agency interventions. One policy was more targeted, a recommendation from NICE that housing assessments should take place where health professionals are concerned that poor indoor air quality may be affecting an individual’s health—though we found no evidence of implementation of this policy in local or national government documents. Active and sustainable travel policies (described above) are also relevant to air pollution.

#### Education policies

The majority of education policies (n=7) were low agency policies, including mandatory school leaving age, free school meals, school transport policies and means-tested financial support for higher and further education. Higher agency policies included outreach interventions to encourage young people from disadvantaged backgrounds to apply for university through myth-busting and mentoring, and local government/school action to address low attendance at school.

### Balance of approach

**Figure 3:**
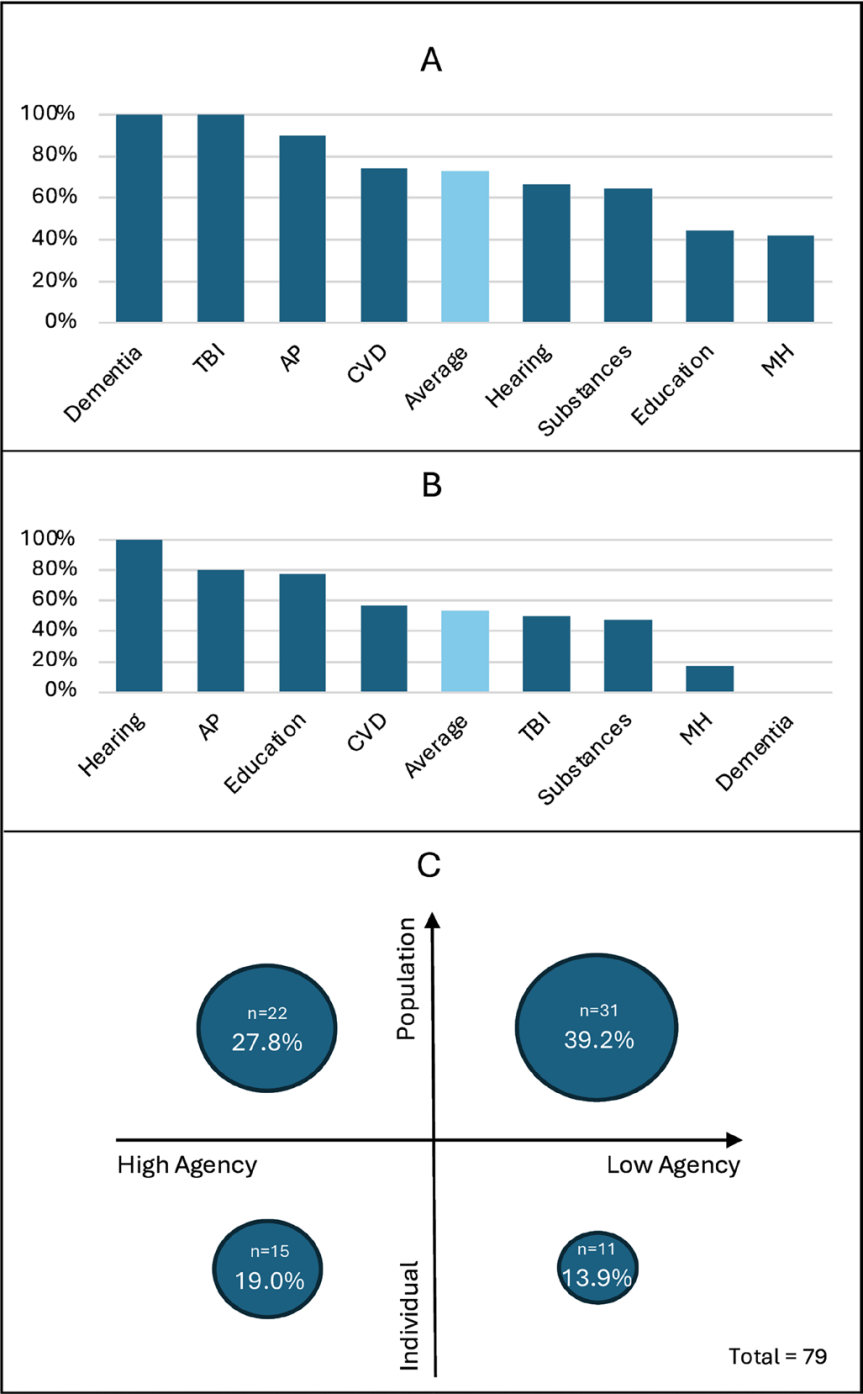
Percentage of current policies that had population-reach, by risk factor (**A**); percentage that required low agency to benefit (**B**), by risk factor; and percentage of current policies that represented each combination of reach (y-axis) and agency (x-axis) (**C**). AP, air pollution; CVD, cardiovascular disease risk factors (eg, hypertension, diabetes); MH, mental health (depression and social isolation); TBI, traumatic brain injury.

Overall, 67.1% (53/79) of current policies had population-reach, 53.2% (42/79) were considered low-agency and 39.2% (31/79) were both population-reach and low-agency (figure 3C).

There was significant variation between risk factors, with high proportions of policies for TBI, air pollution and obesity/physical inactivity/hypertension/diabetes having population-reach; and high proportions of policies for hearing impairment, air pollution and education requiring low-agency to benefit (figure 3).

### Proposed policies

We identified 14 policies which are not yet enacted but have been formally proposed and/or consulted on by organisations listed in figure 1. These are listed in online supplemental table 4 and include policies for obesity/physical inactivity/hypertension/diabetes (n=2), alcohol/smoking (n=6), depression/social isolation (n=1), TBI (n=2) and air pollution (n=3). All but two are proposed by the national government. A higher proportion of these policies meet the criteria for being both population-reach (10/14), low-agency (10/14), and both population-reach and low-agency (8/14), compared with current policies.

## Discussion

### Main findings

In this scoping review using a case study approach to identify dementia primary prevention policies in the UK, we identified 79 distinct, current policies from across national and local tiers of government. We found a balance between policies with population-reach and more targeted measures, and between interventions requiring higher and lower agency in order to benefit from them. The balance differed across risk factors, with some evidence that policies to address depression and social isolation were more likely to have more of an individual-level focus in their reach and require higher-agency to benefit.

Interventions with population-reach, that require low-agency to benefit, are key to reducing dementia incidence equitably.^9^ A higher proportion of the proposed policies, compared with current policies, fulfilled these criteria. However, as such policies are typically more politically contentious,^15^ it may be that they are more likely to be formally proposed in this way before abandoning them after consultation,^24^ while individual-level, higher-agency policies which infringe less on civil liberties may be introduced without a perceived need for formal consultation.^15^

### Findings in context

A 2018 scoping review of English dementia primary prevention policy^18^ reported a patchy and inconsistent policy landscape, but did not explicitly consider the balance of prevention approaches. A recent international review of the prevention components of National Dementia Plans for France, Ireland, Italy, Spain and Sweden^25^ reported a predominant focus on awareness raising (population-reach, high-agency) but a lack of integration with other non-communicable disease policies. In both examples, only policies that were explicitly badged as ‘dementia prevention’ were included, so the majority of relevant policies targeting the risk factors were excluded.

A 2024 review of dementia primary prevention policy across Italian regions^26^ did not include this requirement for dementia prevention to be explicitly listed as the policy driver. The authors did not report on reach or agency specifically, but on four types of end-users: (1) ‘General Population’ (typically awareness raising interventions—population-reach, high-agency); (2) ‘Healthcare Workers’ (typically clinical or lifestyle-based interventions—individual-reach, high-agency); (3) ‘Policymakers’ (typically structural policies—population-reach, low-agency); and (4) ‘Other Stakeholders’ (typically training and partnership work, population-reach, agency unclear). Their findings were consistent with ours, in that they reported a roughly even balance of policies between these groups. As in our study, the authors found relatively few policies related to prevention of hearing loss and TBI, and they also noted a relative shortage of policies addressing public mental health as compared with physical health risk factors.^26^

Considering broader research into disease prevention policy in England, a 2022 review by the Health Foundation examined the reach and agency of national-level policies in England targeting smoking, alcohol, obesity and physical inactivity.^24^ Although the policies identified for these risk factors overlapped with those identified in our review, the authors concluded that national policy showed a trend towards an over-reliance on individual-level and high-agency interventions (whereas we report a balance). This difference in interpretation was partly due to many of the population-reach, low-agency interventions being longstanding, whereas more recent interventions, such as the NHS Better Health campaign, were considered to typically favour a higher-agency approach. Another reason was that, despite being announced as policy, population-level interventions were often then dropped, under-funded or never fully implemented.^24^

### Strengths and limitations

A key strength of our scoping review is the breadth of the search strategy. By combining several data sources, at three tiers of governance, we were able to summarise policies for all 12 of the risk factors identified by the 2020 Lancet Commission report^17^ whether or not they were badged as ‘dementia prevention’.

As is common to many scoping reviews, the trade-off from this breadth is a relative lack of depth. We were not able to meaningfully examine the resourcing, level of implementation and enforcement or evaluation of the policies we identified. Additionally, some policies, such as consideration of public health views in licensing and planning decisions, are enabled by national policy but implemented locally, and assessment of the level of coordination and interactions between different tiers of governance was out of scope for this scoping review exercise.

Some policies are clearly high-agency (eg, lifestyle-based interventions) or low-agency (eg, reformulation programmes). Others (eg, medical and surgical therapies) are more nuanced. Difficulties and inequalities in accessing medical services and adhering to medications are common and well documented,^27 28^ indicating that there is a degree of agency involved. But, on balance, we decided that highly available medical and surgical therapies are more appropriately grouped with other low-agency interventions. There is certainly some subjectivity in these judgements. We considered adopting a framework with three or more levels of agency, but in the absence of an objective, quantitative scale for classifying the level of agency, we decided that the simplicity of the binary classification was preferable.

We were only able to include one region, the East of England, for the regional and local searches. Though the East of England is large and diverse, it may not be representative of the local policy landscape in other English regions.

Lastly, the Lancet Commission published an updated report in summer 2024,^29^ which added visual impairment and high cholesterol to the list of risk factors for which they felt the evidence was sufficient to assert causality. We had already commenced our search strategy at this point and therefore did not consider these additional risk factors. However, we expect that policies for high cholesterol are likely to have mirrored those in the obesity/physical inactivity/hypertension/diabetes group, and those for visual impairment to have some overlap with hearing impairment, so we consider it unlikely that this would have significantly altered our findings.

### Implications for policy

The authors of the Health Foundation review shared our overarching interpretation, rooted in decades of public health theory and evidence,^7 30 31^ that individual-level, high-agency interventions are unlikely to produce the scale of change required to turnaround worsening trends in the prevalence of risk factors such as obesity and physical inactivity, and the stubborn inequalities seen for the harm associated with factors like tobacco and alcohol.^24^ In this sense, it is not a policy ‘balance’ that is required, but a predominance of population-level, low-agency interventions which are coordinated, adequately resourced, and that enjoy broad public acceptability and political support.

In previous work, some authors from this group have synthesised research evidence for ‘population-level interventions’ (population-reach and low-agency) against each of the 12 risk factors listed in the 2020 Lancet commission report, identifying those with strong evidence bases in support.^11^ Considering the current policies identified in this scoping review against these, there are four interventions which could be introduced, and seven others which could be strengthened (table 1). Economic analyses of several of these policies suggest they would be cost-saving through their effects on dementia prevalence.^32^ These include further taxation policies to reduce alcohol and tobacco consumption, enhanced reformulation policies to reduce salt and sugar consumption (to address hypertension and obesity/diabetes risk, respectively), more widespread use of low emission zones to lower air pollution, and legislative change to mandate helmet use by children when cycling, to reduce TBI risk. These require action by both national and local government agencies.

**Table 1:** Comparison of the policy gap between those identified in the present scoping review and those ‘population-level’ (population-reach, low-agency) interventions identified as being supported by high-quality evidence in our previous complex evidence review^11^

Policies not in place that could be introduced	Policies in place that could be strengthened
Policies for obesity, physical inactivity, hypertension and/or diabetes	
Restrictions on volume-based promotions of foods high in fat, sugar, or salt (‘HFSS’) in supermarkets—this was previously proposed but then abandoned by the UK Government	Taxation. For example, increases or broadening of the levy on sugar-sweetened beverages Procurement of healthier food options by public sector organisations (eg, hospital canteens, school meals) Reformulation policies (with clear evidence that mandatory rules are more effective than voluntary agreements often favoured in recent years by government and industry) Urban planning and design policies to increase the accessibility of active travel and active leisure opportunities
Policies for alcohol and tobacco	
Introduce a minimum unit price for alcohol—previously proposed but then abandoned by the UK Government	Taxation. Further increase duty on tobacco and alcohol products Strengthen the role of local public health teams in licensing of the location and operating hours of alcohol venues
Policies for traumatic brain injury	
Mandate the use of helmets for children when using bicycles, with strict enforcement including provision of helmets for those from low-income backgrounds	
Policies for air pollution	
Postponement of non-essential activities (eg, road sweeping) on high pollution days (this could also be achieved by implementing existing ambitions to transfer to emission-free public sector fleets)	Broader implementation of low emission zones and other measures to reduce traffic density in areas of high pollution (high-profile policies have been implemented in major cities such as London and Birmingham). We found no examples of low emission zones in the East of England region

Notwithstanding concerns that the policy trend is towards the individual-level and higher-agency interventions,^24 33^ several ambitious population-reach, low-agency policies have been proposed (online supplemental table 4). These include phased nation-wide bans on the sale of tobacco products and diesel/petrol vehicles, and major advertising and marketing restrictions on unhealthy food and drink products. Staying the course and ensuring these bold public health policies are implemented represent a significant opportunity for the new UK Government.

## Conclusion

Document-based analysis of dementia primary prevention policies in England suggests a balance across axes of reach and agency. However, given the scale, trend and inequity of dementia risk factors, a predominance of population-reach, low-agency policies is required. We identified several policies which could be introduced or strengthened in England to ensure that the policy response is commensurate with the scale of the challenge that dementia poses in the decades ahead.

## Supplementary material

10.1136/bmjph-2025-002631 online supplemental file 1

## Data Availability

All data relevant to the study are included in the article or uploaded as supplementary information.
