# Supplementary material for: Scoping review of dementia primary prevention policies in England: do they balance reach and agency?
Source: BMJ Public Health. 2025 Jun 25;3(1):e002631. doi: 10.1136/bmjph-2025-002631 (PMC12198788; doi:10.1136/bmjph-2025-002631)
Supplement: online supplemental file 1 [file bmjph-3-1-s001.docx]

# Supplementary Materials

Supplementary Table 1 – Preferred Reporting Items for Systematic reviews and Meta-Analyses extension for Scoping Reviews (PRISMA-ScR) Checklist

Supplementary Table 2 – Search Strategy

Supplementary Table 3 – Current dementia primary prevention policies in the UK, grouped by risk factor(s)

Supplementary Table 4 – Proposed dementia primary prevention policies in the UK, grouped by risk factor(s)

Supplementary Table 1 – Preferred Reporting Items for Systematic reviews and Meta-Analyses extension for Scoping Reviews (PRISMA-ScR) Checklist

| **SECTION** | **ITEM** | **PRISMA-ScR CHECKLIST ITEM** | **REPORTED ON PAGE #** |
| --- | --- | --- | --- |
| **TITLE** | | | |
| Title | 1 | Identify the report as a scoping review. | Title |
| **ABSTRACT** | | | |
| Structured summary | 2 | Provide a structured summary that includes (as applicable): background, objectives, eligibility criteria, sources of evidence, charting methods, results, and conclusions that relate to the review questions and objectives. | Abstract |
| **INTRODUCTION** | | | |
| Rationale | 3 | Describe the rationale for the review in the context of what is already known. Explain why the review questions/objectives lend themselves to a scoping review approach. | Introduction |
| Objectives | 4 | Provide an explicit statement of the questions and objectives being addressed with reference to their key elements (e.g., population or participants, concepts, and context) or other relevant key elements used to conceptualize the review questions and/or objectives. | Introduction |
| **METHODS** | | | |
| Protocol and registration | 5 | Indicate whether a review protocol exists; state if and where it can be accessed (e.g., a Web address); and if available, provide registration information, including the registration number. | Methods |
| Eligibility criteria | 6 | Specify characteristics of the sources of evidence used as eligibility criteria (e.g., years considered, language, and publication status), and provide a rationale. | Methods |
| Information sources* | 7 | Describe all information sources in the search (e.g., databases with dates of coverage and contact with authors to identify additional sources), as well as the date the most recent search was executed. | Methods |
| Search | 8 | Present the full electronic search strategy for at least 1 database, including any limits used, such that it could be repeated. | Methods + Appendix 1 |
| Selection of sources of evidence† | 9 | State the process for selecting sources of evidence (i.e., screening and eligibility) included in the scoping review. | Methods |
| Data charting process‡ | 10 | Describe the methods of charting data from the included sources of evidence (e.g., calibrated forms or forms that have been tested by the team before their use, and whether data charting was done independently or in duplicate) and any processes for obtaining and confirming data from investigators. | Methods |
| Data items | 11 | List and define all variables for which data were sought and any assumptions and simplifications made. | Methods |
| Critical appraisal of individual sources of evidence§ | 12 | If done, provide a rationale for conducting a critical appraisal of included sources of evidence; describe the methods used and how this information was used in any data synthesis (if appropriate). | Methods |
| Synthesis of results | 13 | Describe the methods of handling and summarizing the data that were charted. | Methods |
| **RESULTS** | | | |
| Selection of sources of evidence | 14 | Give numbers of sources of evidence screened, assessed for eligibility, and included in the review, with reasons for exclusions at each stage, ideally using a flow diagram. | Results |
| Characteristics of sources of evidence | 15 | For each source of evidence, present characteristics for which data were charted and provide the citations. | Results, Tables 1 and 2, + Full Extraction Tables (available on request) |
| Critical appraisal within sources of evidence | 16 | If done, present data on critical appraisal of included sources of evidence (see item 12). | n/a |
| Results of individual sources of evidence | 17 | For each included source of evidence, present the relevant data that were charted that relate to the review questions and objectives. | Results, Tables 1 and 2, + Full Extraction Tables (available on request) |
| Synthesis of results | 18 | Summarize and/or present the charting results as they relate to the review questions and objectives. | Results |
| **DISCUSSION** | | | |
| Summary of evidence | 19 | Summarize the main results (including an overview of concepts, themes, and types of evidence available), link to the review questions and objectives, and consider the relevance to key groups. | Discussion |
| Limitations | 20 | Discuss the limitations of the scoping review process. | Discussion |
| Conclusions | 21 | Provide a general interpretation of the results with respect to the review questions and objectives, as well as potential implications and/or next steps. | Discussion |
| **FUNDING** | | | |
| Funding | 22 | Describe sources of funding for the included sources of evidence, as well as sources of funding for the scoping review. Describe the role of the funders of the scoping review. | Funding |

| Supplementary Table 2 – Search Strategy | |
| --- | --- |
| **Database Searches** | |
| Medline via Ovid | *Ovid MEDLINE(R) and Epub Ahead of Print, In-Process, In-Data-Review & Other Non-Indexed Citations, Daily and Versions <1946 to June 10, 2024>*  1 (dementia or alzheimer* or cognitive impariment or physical inactivity or physical activity or obesity or overweight or healthy diet or unhealthy diet or sugar sweetened beverages or sugary drinks or diabet* or hypertension or high blood pressure or (salt adj2 (reduction or intake or consumption)) or smok* or tobacco or alcohol* or (air adj3 pollut*) or head injur* or brain injur* or social isolation or socially isolated or lonely or loneliness or depression or (public adj3 mental health) or educat* or school* or hearing impairment or hearing loss or deaf* or hearing aid*).ti,ab. 3921214  2 (policy or policies or guidance or guideline* or strategy or strategic plan or action plan*).ti. 274674  3 (intervention or prevent* or reduc* or control or increas* or decreas* or improve or protect* or promot*).ti,ab. 14568296  4 exp United Kingdom/ or (english not ((published or publication* or translat* or written or language* or speak* or literature or citation*) adj5 english)).ti,ab. or (gb or "g.b." or britain* or (british* not "british columbia") or uk or "u.k." or united kingdom* or (england* not "new england")).ti,ab,jw,in. or (national health service* or nhs* or "national institute for health and care excellence" or "NICE" or Public Health England or "PHE" or uk health security agency or UKHSA or government*).ti,ab,in. 3030604  5 (integrated care board or integrated care system or integrated care partnership or "ICS" or clinical commissioning group or "CCG" or primary care or general practice or community mental health or local government or local authority or regional authority or combined authority or unitary authority or council or "health and wellbeing board" or HWB or locality).ti,ab,in. 471572  6 (East of England or Cambridgeshire or Peterborough or Norfolk or Suffolk or Hertfordshire or Herts or Bedfordshire or Beds or Luton or Essex or Southend or Thurrock or (cambridge not (massachusetts* or boston* or harvard*)) or Norwich or Ipswich or Bedford or Broxbourne or Dacorum or Hertsmere or St Albans or Saint Albans or Stevenage or Three Rivers or Watford or Welwyn Hatfield).ti,ab,in. 306200  7 5 and 6 21096  8 4 or 7 3032812  9 1 and 2 and 3 and 8 5924  10 limit 9 to yr="2019 -Current" 2563 |
| HMIC via Ovid | *HMIC Health Management Information Consortium <1979 to March 2024>*  1 (dementia or alzheimer* or cognitive impariment or physical inactivity or physical activity or obesity or overweight or healthy diet or unhealthy diet or sugar sweetened beverages or sugary drinks or diabet* or hypertension or high blood pressure or (salt adj2 (reduction or intake or consumption)) or smok* or tobacco or alcohol* or (air adj3 pollut*) or head injur* or brain injur* or social isolation or socially isolated or lonely or loneliness or depression or (public adj3 mental health) or educat* or school* or hearing impairment or hearing loss or deaf* or hearing aid*).mp. [mp=title, other title, abstract, heading words] 71603  2 (policy or policies or guidance or guideline* or strategy or strategic plan or action plan*).mp. [mp=title, other title, abstract, heading words] 75501  3 (United Kingdom or UK or Great Britain or British or England or English or NHS or National Health Service or "National Institute for Health and Care Excellence" or NICE or Public Health England or PHE or Health Security Agency or UKHSA).mp. [mp=title, other title, abstract, heading words] 129602  4 (integrated care board or integrated care system or integrated care partnership or "ICS" or clinical commissioning group or "CCG" or primary care or general practice or community mental health or local government or local authority or regional authority or combined authority or unitary authority or council or "health and wellbeing board" or HWB or locality).mp. [mp=title, other title, abstract, heading words] 50811  5 (East of England or Cambridgeshire or Peterborough or Norfolk or Suffolk or Hertfordshire or Herts or Bedfordshire or Beds or Luton or Essex or Southend or Thurrock or (cambridge not (massachusetts* or boston* or harvard*)) or Norwich or Ipswich or Bedford or Broxbourne or Dacorum or Hertsmere or St Albans or Saint Albans or Stevenage or Three Rivers or Watford or Welwyn Hatfield).mp. [mp=title, other title, abstract, heading words] 5937  6 4 and 5 1021  7 3 or 6 130108  8 1 and 2 and 7 5060  9 limit 8 to yr="2019 -Current" 420 |
| Overton | *12/06/24, filters: published after 01/06/2019, from UK, not from places outside of case study area (e.g. Scotland, Ireland, Wales, Manchester, Birmingham – selectively excluded authors from these areas if they had published more than 100 articles meeting other criteria)*  (abstract: (dementia OR alzheimer* OR “cognitive impairment” OR “physical inactivity” OR “physical activity” OR obesity OR overweight OR “healthy diet” OR “unhealthy diet” OR “sugar sweetened beverages” OR “sugary drinks” OR diabet* OR hypertension OR “high blood pressure” OR “salt reduction”~2 OR “salt intake”~2 OR “salt consumption”~2 OR smok* OR tobacco OR alcohol* OR “air pollut*”~3 OR “head injur*” OR “brain injur*” OR “social isolation” OR “socially isolated” OR lonely OR loneliness OR depression OR “mental health” OR educat* OR school* OR “hearing impairment” OR “hearing loss” OR deaf* OR “hearing aid*”))  AND  (title: (policy OR policies OR guidance OR guideline* OR strategy OR strategic plan OR action plan*))  AND  (abstract: (intervention OR prevent* OR reduc* OR control OR increas* OR decreas* OR improve OR protect* OR promot*))  AND  ((abstract: (“United Kingdom” OR UK OR “Great Britain” OR British OR England OR English OR NHS OR “National Health Service” OR “National Institute for Health and Care Excellence” OR NICE OR “Public Health England” OR PHE OR “Health Security Agency” OR UKHSA)) OR ((Abstract: (“Integrated Care Board” OR “Integrated Care System” OR “Integrated Care Partnership” OR ICS OR ICB OR ICP OR “Clinical Commissioning Group” OR CCG OR “Primary Care” OR “General Practice” OR “Community Health”~2 OR “Local Government” OR “Local Authority” OR “Regional Authority” OR “Combined Authority” OR “Unitary Authority” OR Council OR “Health and Wellbeing Board” OR HWB OR Locality)) AND (Abstract: (“East of England” OR Cambridgeshire OR Peterborough OR Norfolk OR Suffolk OR Hertfordshire OR Herts OR Bedfordshire OR Beds OR Luton OR Essex OR Southend OR Thurrock OR Cambridge OR Norwich OR Ipswich OR Bedford OR Broxbourne OR Dacorum OR Hertsmere OR St Albans OR Saint Albans OR Stevenage OR Three Rivers OR Watford OR “Welwyn Hatfield”))))  N=1617 hits |
| **Web Searches** | |
| Gov.uk | Search date 13/06/24  Searched filters: Guidance and Regulation, Services, Policy Papers, Consultations  Limits:2019-current  Searches:   1. Dementia (n=45) 2. Education. Sub-topics: education of disadvantaged children (n=20), funding and finance for children (n=51), further and higher education, skills and vocational training (n=326), pupil wellbeing, behaviour and attendance (n=45), and starting and attending school (n=6) 3. Hearing loss. Topics: Business and industry (n=19), health and social care (n=30), work (n=8) 4. Brain head injury. Topics: Health and social care (n=11), society and culture (n=1), transport (n=11), work (n=2) 5. Hypertension - health and social care (n=6); 'salt' - business and industry (n=13), health and social care (n=11); 'blood Pressure' - business and industry (n=2), health and scial care (n=15). 6. Alcohol - business and industry, business regulation (n=17), manufacturing (n=2); health and social care (n=58); regional and local government (n=1); society and culture (n=3) 7. Obesity (n=24), 'diet' - health and social care (n=23); 'sugar' - business and industry, business regulation (n=3); health and social care (n=13) 8. Tobacco - business and industry, business regulation (n=4); health and social care, NHS (n=3), public health (n=28); money, business tax (n=55); 'smoking' - health and social care, public health (n=40) 9. Depression (n=18). 'mental health' - policy documents only; health and social care, public health (n=46) 10. Social isolation - health and social care, public health (n=27); housing, local and community (n=11), society and culture (n=15), transport (n=2) 11. Phsyical inactivity - education, training and skills, running and managing a school (n=10), school and academy funding (n=10), school curriculum (n=19); health and social care, public health (n=150); society and culture, sports and leisure (n=12) 12. Air pollution - business and industry, business regulation (n=35); environment, pollution and environmental quality (n=104), health and social care, public health (n=42); housing, local and community, household energy (n=1), housing and communities (n=22)); regional and local government (n=5); transport (n=310) 13. Diabetes - health and social care (n=82) |
| UKHSA | UK Health Security Agency  Search date: 14/06/24  Manual navigation through website to review any policy documents (n=12) |
| NHS England | Search date: 14/06/24  Search filters: Publications  Searches:   1. General - 'long term plan' (n=2) 2. 'Dementia' (n=13) 3. Education - not searched as not relevant to NHSE role 4. 'Hearing ' (n=4). Deaf (n=0) 5. 'Brain injury' (n=0); 'Head injury' (n=0) 6. 'Hypertension' (n=1), 'blood pressure' (n=1), 'salt' (n=0) 7. 'Alcohol' (n=3) 8. 'Obesity' (n=0), 'Overweight' (n=0), 'Weight' (n=12), 'Diet' (n=2), 'Sugar' (n=0) 9. 'Tobacco' (n=1), 'Smoking' (n=2) 10. 'depression' (n=3). 11. 'social isolation' (n=1), 'lonely (n=0), 'loneliness' (n=0) 12. 'physically active' (n=0), 'physically inactive' (n=0), 'inactive (n=0), 'active' (n=5) 13. 'air pollution' (n=0), 'pollution' (n=0), added from gov.uk searches (n=1) 14. 'diabetes' (n=15) |
| NICE | National Institute for Health and Care Excellence  Search date: 14/06/24  Search filters: Published guidance  No date limits as can only limit to a max of 3 yrs ago  Sorted by relevance, first 30 results screened  Searches:   1. Dementia (n=63) 2. Education - not searched as not relevant to NHSE role 3. Hearing loss (n=50) 4. Brain injury (n=59), head injury (n=63) 5. Hypertension (n=186), salt (n=23) 6. Alcohol (n=161) 7. Obesity (n=160) 8. Smoking (n=133) 9. Depression (n=219) 10. Social Isolation (n=85), loneliness (n=10) 11. Physical Inactivity (n=13) 12. 'Air pollution' (n=10) 13. 'Diabetes' (n= 231) |
| Upper Tier Authorities | *Manual navigation through website to identify relevant departments, policies and service webpages. If no relevant page(s) identified then search function used as appropriate.*  Cambridgeshire County Council (search date: 16/06/24)   1. 'dementia' – search (n=24) 2. 'education' - policy page identified - 'key strategies and plans' and pages (n=6) 3. 'hearing loss' – search (n=20) 4. 'head injury' (n=3), 'brain injury' (n=6), 'helmet' (n=3) - search 5. 'hypertension' (n=0), 'blood pressure (n=6), 'salt' (n=4); policy page – 1 6. 'alcohol' (n=26); policy page – 1 7. 'obesity' (n=4), healthy weight (n=7), diet (n=11); policy page – 1 8. 'smoking' (n=17); policy page -1 9. 'depression' (n=13); policy page -1 10. 'social isolation' (n=24) 11. 'physical inactivity' (n=1), 'physical activity' (n=59); policy page (n=2) 12. 'air pollution' (n=27) 13. 'diabetes' (n=6)   Essex County Council (search date: 10/07/24)   1. 'dementia' - no policy available via the policy or dementia pages on website. Search 'dementia' (n=26) 2. 'education' - section on policy page (n=8) 3. 'hearing loss' - sensory impairments page n=0 policies, policy page n=0, search 'hearing loss (n=105) 4. 'head injury' – search (n=52) 5. 'hypertension' (n=0), 'blood pressure' (n=41), salt (n=2), policy page (n=1) 6. 'alcohol' (n=21) 7. 'obesity' (n=2), 'diet' (n=4), policy page - no new relevant policies (see above) 8. 'smoking' (n=9) 9. 'depression' (n=7), policy page (n=1) 10. 'social isolation' - 'loneliness' (n=4) 11. 'physical inactivity' (n=89), policy page (n=1) 12. 'air pollution', policy page travel (n=1), environment (n=2) 13. 'diabetes' (n=7)   Herts County Council (search date: 10/07/24)   1. 'dementia' -main policy page (n=1) 2. 'education' (n=12), 'school' (n=7) - both main policy page 3. 'hearing loss' . Main policy page: hearing loss, hearing impairment, deaf (n=0), sensory services policy page (n=1) 4. 'head injury'. Main policy page: head injury, brain injury (n=0); road safety policy page (n=1) 5. 'hypertension'. Main policy page: hypertension, blood pressure, salt (n=0); general search (n=38) 6. 'alcohol'. Main policy page (n=1) 7. 'obesity'. Main policy page: obesity, weight, diet, eating (n=0); public health policy pages (n=1); general search 'obesity' (n=6), 'diet' (n=22) 8. 'smoking'. Main policy page: smoking, smoke, tobaco (n=0); public health page (n=1); stop smoking page 9. 'depression'. Main policy page: 'depression' (n=0), 'mental health' (n=5) 10. 'social isolation'. Main policy page: 'social isolation' 'lonely' 'loneliness' (n=0). General search: 'loneliness' (n=6) 11. 'physical inactivity'. Main policy page: 'physical inactivity' 'physical activity' (n=0); health pages (n=3) 12. 'air pollution'. Main policy page: 'pollution' (n=0), 'climate' (n=1). Environmental policy page (n=1) 13. 'diabetes'. General search (n=16)   Norfolk County Council (search date: 11/07/24)   1. 'dementia' - main policy page (n=1); search 'dementia' (n=28) 2. 'education' - main policy page (n=6) 3. 'hearing loss' . Hearing impairment specific page (n=1) 4. 'head injury'. Main policy page (n=1), Road safety page (n=1) 5. 'hypertension' . Main policy page (n=3). Public health pages (n=1). 6. 'alcohol'. Public health pages (n=2) 7. 'obesity'. Main policy page (n=2), public health pages (n=1) 8. 'smoking'. Public health pages (n=1) 9. 'depression' . Public health pages (n=3) 10. 'social isolation'. Main policy page (n=1), general search 'loneliness' (n=7) 11. 'physical inactivity' . Main policy page (n=5) 12. 'air pollution'. Main policy page (n=5) 13. 'diabetes' . Public health page (n=1)   Suffolk County Council (search date: 11/07/24)   1. 'dementia' - main policy page (n=6), 'dementia' general search (n=13) 2. 'education' - Main policy page (n=1), Education page (n=3) 3. 'hearing loss' - search 'sensory impairment' n=4, 'hearing impairment' n=6, 'hearing loss' n=7, 'deaf' n=2 4. 'head injury' - Policy page n=1, 'head injury' n=6, 'brain injury' n=0, 'helmet' n=3 5. 'hypertension' - 'hypertension' n=0, 'blood pressure' n=2 6. 'alcohol' - 'alcohol' n=21 7. 'obesity' - 'obesity' n=4 8. 'smoking' - 'smoking' n=25 9. 'depression' (n=10) 10. 'social isolation' - 'loneliness' (n=7) 11. 'physical inactivity' - main policy page (n=2), public health page (n=1) 12. 'air pollution' - main policy page (n=1),'air pollution' (n=7) 13. 'diabetes' (n=5) |

| Unitary Authorities | *Manual navigation through website to identify relevant departments, policies and service webpages. If no relevant page(s) identified then search function used as appropriate.*  Peterborough Council (search date: 12/07/24)  *Main strategies, priorities and plans page. Health and social care pages. And general search*   1. 'dementia' - 'dementia' (n=3) 2. 'education' - Main policy page (n=1), Education pages (n=5) 3. 'hearing loss' - search 'sensory impairment' n=8, 'hearing impairment' 4. 'head injury' - Road safety pages (n=1). Search 'helmet' (n=11) 5. 'hypertension' - main policy page (n=1), 'hypertension' n=0, 'blood pressure' n=6, food standards pages (n=1) 6. 'alcohol' - 'alcohol' n=45 7. 'obesity' - 'obesity' n=2, 'diet' n=8 8. 'smoking' (n=9) 9. 'depression'. Main policy page (n=1) 10. 'social isolation' - 'loneliness' (n=17) 11. 'physical inactivity' - main policy page (n=2) 12. 'air pollution' - environmental health page (n=3) 13. 'diabetes' (n=14)   Southend Council (15/07/24)  *No central policy page so reviewed relevant landing pages via site map and general search where appropriate*   1. 'dementia' - site map naviagation (n=1) 2. 'education' - education policies, plans, and strategies page (n=3), JSNAs (n=1) 3. 'hearing loss' - physical and senosry impairments page (n=1) 4. 'head injury' - road safety page (n=1), 'brain injury' (n=7) 5. 'hypertension' - health and social care pages 6. 'alcohol' - health and social care pages 7. 'obesity' - health and social care pages (n=0), 'diet' search (n=17) 8. 'smoking' - health and social care pages 9. 'depression' - health and social care pages 10. 'social isolation' - 'loneliness' n=18 11. 'physical inactivity' - health and social care pages (n=1), sports and leisure pages via site map (n=1), transport policies via sitemap (n=1) 12. 'air pollution' - annual report from site map (n=1), environmental pages via sitemap (n=4) 13. 'diabetes' - health and social care pages   Thurrock Council (15/07/24)  *Priorities and strategies page. Otherwise identified landing page from site map at base of homepage, general search as appropriate*   1. 'dementia' - main policy page (n=2), health pages (n=2) 2. 'education' - main policy page (n=1), education pages (n=3) 3. 'hearing loss' - adult care and health pages (n=1) 4. 'head injury' - road safety page (n=1) 5. 'hypertension' - Adult health pages (n=3) 6. 'alcohol' - Adults health pages (n=2) 7. 'obesity' -Adults health pages (n=4) 8. 'smoking' - Adults health pages (n=3) 9. 'depression' - Adults health pages (n=2) 10. 'social isolation' - 'loneliness (n=1) 11. 'physical inactivity' - Adult health pages (n=2), Education pages (n=1) 12. 'air pollution' - travel strategies (n=1), environment pages (n=2) 13. 'diabetes' - Adult health pages (n=0), 'Diabetes' (n=12)   Luton Council (15/07/24)  *Central policy page, then other landing pages accessed from home page, then general search as appropriate*   1. 'dementia' - main policy page (n=2), health and social care pages (n=2) 2. 'education' - education pages (n=3) 3. 'hearing loss' - health and social care pages (n=1) 4. 'head injury' - transport pages (n=4) 5. 'hypertension' - Health and social care pages (n=4) 6. 'alcohol' - Health and social care pages (n=1) 7. 'obesity' - health and social care pages (n=3) 8. 'smoking' - health and social care pages (n=2) 9. 'depression' - health and social care pages (n=2) 10. 'social isolation' – search (n=0) 11. 'physical inactivity' - health and social care pages (n=5), main policy page (n=1), transport pages (n=6) 12. 'air pollution' - main policy page (n=1), environment pages (n=6) 13. 'diabetes' - search (n=0)   Bedford Borough (15/07/24)  *Main policy page. Otherwise reviewed relevant landing pages. General website search as appropriate*   1. 'dementia' - social care and health pages (n=3) 2. 'education' - main policy page (n=2) 3. 'hearing loss' - social care and health pages (n=1) 4. 'head injury' - transport pages (n=3) 5. 'hypertension' - social care and health pages (n=2) 6. 'alcohol' - Social care and health pages (n=2), trading standards (n=1) 7. 'obesity' - social care and health pages (n=3) 8. 'smoking' - Social care and health pages (n=1) 9. 'depression' - social care and health pages (n=2) 10. 'social isolation' – search (n=0) 11. 'physical inactivity' - Main policy page (n=1), social care and health pages (n=1), transport pages (n=4), planning pages (n=1) 12. 'air pollution' - environmental pages (n=3) 13. 'diabetes' - search (n=0)   Central Beds (15/07/24)  *No central policy page so navigated to sector specific pages, general search where appropriate*   1. 'dementia' - about your council pages (n=2), health and social care pages (n=1) 2. 'education' - children and young people's pages (n=1), education pages (n=4) 3. 'hearing loss' - health and social care pages (n=1) 4. 'head injury' - transport pages (n=1) 5. 'hypertension' - health and social care pages (n=4) 6. 'alcohol' - health and social care pages (n=1), trading standards (n=1) 7. 'obesity' - health and social care pages (n=1) 8. 'smoking' - health and social care pages (n=1) 9. 'depression' - health and social care pages (n=1) 10. 'social isolation' - health and social care pages (n=2) 11. 'physical inactivity' - transport pages (n=1), planning pages (n=1), education pages (n=1), leisure pages (n=2) 12. 'air pollution' - environment pages (n=3), education pages (n=1) 13. 'diabetes' - search (n=0) |
| --- | --- |
| HWBs | Health and Wellbeing Boards  All searched 16/07/24  *Accessed via the health and wellbeing board webpages (housed via the relevant council and ICS websites)*  Cambridgeshire and Peterborough HWB – Integrated Care Strategy (n=1)  Essex HWB – Essex (n=1), Thurrock (n=1), Southend (n=1)  Hertfordshire HWB – Health and Wellbeing Strategy (n=1)  Norfolk HWB – Health and Wellbeing Strategy (n=1), Healthy Lifestyles and Behaviour Change – A Systems Approach (n=1)  Suffolk HWB - Preparing for the Future. Joint Local Health and Wellbeing Strategy (n=1)  Bedfordshire and Luton HWB – Beds Borough (n=0), Central Beds (n=0), Luton (n=0) |
| ICSs | Integrated Care Systems  All searched 16/07/24  *Documents extracted from ICS websites policy/strategy/document pages, otherwise via manual navigation of websites to identify potentially relevant policies (smaller websites as these are newer organisations with a narrower remit compared to local authorities, so easier to identify relevant policies)*  Cambridgeshire and Peterborough ICS   - Health and Wellbeing and Integrated Care Strategy and associated documents (n=4) - Joint Forward Plan (n=1) - Green plan (n=1)   Mid and South Essex ICS   - Integrated care strategy (n=1) - Joint forward plan (n=1) - Green plan (n=1)   Herts and West Essex ICS   - Integrated care strategy (n=1) - Joint forward plan (n=1) - Green plan (n=1) - Healthcare strategies (n=3)   Norfolk and Waveney ICS   - Integrated Care and Health and Wellbeing Strategy (n=1) - Joint Forward Plan (n=1) - Clinical Strategy (n=1) - Health Inequalities Strategic Framework for Action (n=1) - Approach To Working With People and Communities (n=1) - Green Plan (n=1)   Suffolk and North East Essex ICS   - Integrated Care Strategy (n=1) - Joint Forward Plan (n=1) - Guide to PHM (n=1) - Green Plan (n=1)   Bedfordshire, Luton, and Milton Keynes ICS   - Integrated care strategy (n=1) - Joint forward plan (n=1) - PHM strategy (n=1) - People and community strategy (n=1) - Long-term plan (n=1) - Green plan (n=1) |
| Combined Authority | Cambridgeshire and Peterborough (search date: 16/07/24)  *Manual navigation through website to identify relevant policies and strategies*   - Business Plan (n=1) |
| Herts District Councils | All searched 16/07/24  *Identify policy pages, where available, then navigate to relevant parts of websites to identify other policies/services*  Broxbourne Borough Council   - Corporate Plan (n=1) - Arts, Culture & Heritage Strategy (n=1) - Local Plan (n=1) - Sustainability Strategy and Action Plan (n=1) - Licensing Policy (n=1) - Healthy Hub Broxbourne (n=1)   Dacorum Borough Council   - Corporate Plan (n=1) - Growth and Infrastructure Strategy (n=1) - Physical Activity and Sports Strategy (n=1) - Local Plan (n=1) - Core Strategy (n=1) - Climate and Ecological Emergency Strategy (n=1) - Electric Vehicle Strategy (n=1)   East Herts Council   - Corporate Plan (n=1) - Local Plan (n=1) - Healthy Hub East Herts (n=1) - Forever Active East Herts (n=1) - Air Quality Action Plan (n=1) - Climate Change Strategy (n=1) - Workplace Safety (includes noise exposure) (n=1)   Hertsmere Council   - Corporate Plan (n=1) - Climate Change and Sustainability Strategy (n=1) - Physical Activity and Wellbeing Strategy (n=1) - Health Hub (n=1) - Local Plan (2024- doc in draft) (n=1)   North Herts Council   - Corporate Plan (n=1) - Climate Change Strategy (n=1) - Greenspace Management Strategy (n=1) - Local Plan (n=1) - Health Hub (n=1)   St Albans District Council   - Council Plan (n=1) - Sustainability and Climate Crisis Strategy (n=1) - Current Local Plan (in development) (n=1) - Healthy Hub (n=1)   Stevenage Council   - Making Stevenage Better (Council Plan) (n=1) - Co-operative Commitment (strategy for working with communities) (n=1) - Climate Change Strategy (n=1) - Local Plan (n=1) - Culture Strategy (n=1) - Healthy Stevenage Position Statement (n=1) - Healthy Hub (n=1) - Air Quality Strategy (n=1)   Three Rivers District Council   - Corporate Strategy (n=1) - Climate Emergency and Sustainability Strategy (n=1) - Community Strategy (n=1) - Sport and Physical Activity Strategy (n=1) - Green Travel Plan (n=1) - Local Plan (new one in development) (n=1) - Healthy hubs (n=1)   Watford Borough Council   - Council Plan (n=2) - Local Plan (n=1) - Economic Growth Strategy (n=1) - Cultural Strategy (n=1) - Green Spaces Strategy (n=1) - Sports Facilities Strategy (n=1) - Playing Pitch Strategy and Action Plan (n=1) - Climate Change Strategy (n=1)   Welwyn Hatfield Borough Council   - Corporate Plan (n=1) - Transformation Strategy (n=1) - Community and Engagement Strategy (n=1) - Climate Change Strategy (n=1) - Local Plan (n=1) - Playing Pitch Strategy (n=1) - Built Facilities Strategy (n=1) |

| Supplementary Table 3 – Current dementia primary prevention policies in the UK, grouped by risk factor(s) | | | |
| --- | --- | --- | --- |
| Policy/Intervention | Lead org(s) | Reach | Agency |
| **Policies with dementia risk reduction as an explicit aim** |  |  |  |
| NHS Health Checks (see below*). Those >65 receive dementia risk reduction messaging, including a leaflet outlining modifiable risk factors | NHS, Local gov | Pop | High |
| Give individuals information about reducing their personal risk of getting dementia in order to minimise the risk of people developing dementia. Incorporate dementia risk reduction messaging into MECC Programmes**, including the 'What's good for your heart is good for your brain' messaging | NHS | Pop | High |
| Raise awareness of dementia risk factors and the possibility of risk reduction through national, regional, and local campaigns aimed at the public and health professionals | UK gov, Local gov | Pop | High |
| **Policies for obesity, physical inactivity, hypertension, and/or diabetes** |  |  |  |
| Food reformulation programmes. Soft Drinks Industry Levy is a tiered mandatory levy on sugar sweetened beverages, implemented in 2018. Other reformulation 'challenges' on salt, sugar, and calories are voluntary (but do have evidence of some impact) | UK gov | Pop | Low |
| Restrictions, financial incentives, nutritional standards, directives, and award schemes for Gov agencies, NHS premises, local workplaces, and schools to offer healthier food options, including limiting the availability, proportion, placement, and/or promotion of foods and beverages high in fat, salt, and/or sugar; and provision of shower facilities to encourage active travel to work | UK gov, NHS, Local gov | Pop | Low |
| Planning decisions that make healthy choices easier e.g. limiting new takeaways in areas of high childhood obesity, increasing active travel routes and green space access, prioritisation of active travel infrastructure (e.g. cycle lanes) over car transport when approving new developments and highway improvements (N.B. these activities are linked in national documents explicitly to dementia risk reduction - see sources) **^^** | Local gov | Pop | Low |
| Funding for improvements and expansions of walking and cycling route infrastructure, green spaces, leisure centres and sports facilities - sometimes with particular targeting of active school travel/commuting, and communities where PI rates are highest. 'Green flag' accreditation scheme for high-quality parks and open spaces **^, ^^** | UK gov; local gov | Pop | Low |
| Road policies to encourage active travel: including implementation of 20mph zones in areas with vulnerable road users and areas that would benefit from more activity travel; 'School Streets' policies which impose temporary restrictions on motorised traffic at pick-up and drop-off times outside of schools; 'School Crossing Patrols' (lollipop men/women), 'Low Traffic Neighbourhoods' (pedestrianised road sections). Requirement for schools, and support for workplaces, to develop and implement 'travel plans' which address barriers and encourage active travel to school/work - with national accreditation through ModeShift STARS programme **^, ^^** | UK gov, Local gov | Pop | Low |
| Provide free/subsidised cycling training and equipment - for example 'Bikeability' training in schools and Cycle to Work schemes e.g. Essex Pedal Power (free bikes for NHS staff bands 2-4) **^^** | UK gov; Local gov | Pop | Low |
| Mandatory calorie labelling of non-pre-packaged food in larger out-of-home food sector businesses (voluntary for smaller businesses). Mandatory nutritional reporting on back of pre-packaged food in supermarkets, with voluntary front of packet traffic light labelling | UK gov | Pop | Low |
| Restrictions on locations of foods high in fat, salt and sugar in large shops and online retailers (policy on restrictions of volume-based promotions dropped, citing 'cost of living crisis') | UK gov | Pop | Low |
| Advertising restrictions for products high in fat, salt, and/or sugar during children’s TV and other programming of particular appeal to children | UK gov | Pop | Low |
| Minimum requirement of, and dedicated funding for, at least 2 hours of physical education per week in schools, and active processes to ensure equity of access | UK gov | Pop | Low |
| *NHS Health Checks. Offered by GPs, pharmacies, community health hubs every 5 years to people 40-74 without known chronic disease. Measure BMI, BP, cholesterol, glucose, and lifestyle questions; refer to primary care or lifestyle services. Policy drives to increase uptake, particularly in more deprived areas | NHS | Pop | High |
| Mini health checks' at community venues e.g. libraries, pharmacies, GP surgeries, usually open to all adults, offering things like BP, BMI, and heart rate checks with onward signposting | Local gov, NHS | Pop | High |
| Outreach public health support to workplaces to deliver health checks, healthy lifestyle advice, support to establish health champions and workplace health challenges, and signposting (e.g. to active commuting support) | Local gov | Pop | High |
| NHS standard contract mandates providers to maintain organisational plans for delivering MECC**; 'health coaching' motivational interviewing offers in primary care; and Local gov provide training for local professional groups | NHS, Local gov | Pop | High |
| National and local media campaigns such as 'Better Health', 'Change4Life', 'This Girl Can', 'Eat well, Feel Well', 'Move More Autumn Challenge' and healthy lifestyle information. Include adverts, collections of information and advice, risk scores, links to NHS-approved digital health apps, signposting to local leisure services, exercise groups, lessons and routes, advice, and digitally interactive challenges for individuals wanting to lose weight, eat healthier, and/or get active including modal shifts to active/sustainable travel options **^^** | UK gov, NHS, Local gov | Pop | High |
| Mandatory teaching in school about the benefits of physical exercise, and what constitutes a healthy diet. Voluntary health schools rating scheme in which schools receive awards based on their self-assessment | UK gov | Pop | High |
| Signposting to, and support for, social prescribing schemes and volunteer based activities like Park Run, conservation groups, allotment schemes, walking groups, and cookery classes - which are sometimes free to attend | Local gov | Pop | High |
| Offer medical treatment to all patients with diabetes and hypertension. Specialised services offering medications and bariatric surgery for those with (morbid) obesity and type 2 diabetes or hypertension, and those who do not benefit from lifestyle programmes. Policy drives to improve management pathways (e.g. using home monitoring, Population Health Management), and reduce health inequalities (e.g. CORE20PLUS5) | NHS | Ind | Low |
| Means-tested programmes run nationally and administered locally, e.g. 'Holiday Activities and Food Programme', providing children from low income families free healthy meals and access to physical activity during school holidays; and 'Healthy Start Scheme', providing low-income pregnant women and young mothers with pre-paid cards for fruit and vegetable purchases | UK gov, Local gov | Ind | Low |
| Working with local businesses to increase occupational opportunities, including apprenticeships and pathways to employment, across the board but particularly for those who are (or are at risk of) NEET, and those with learning disability and autism - on the basis that this is a key upstream driver of lifestyle choices such as diet | Local gov | Ind | Low |
| Community-based healthy lifestyle services offering free weight management support through dietary counselling (through 1:1 or group; face-to-face or virtual and digital apps), typically 12 week duration; and advice/financial support for getting active including 'exercise on referral' schemes. Self- or professional-referral. Sometimes situated in community integrated 'health hubs'. Sometimes with a specific offer for overweight children and their families, pregnant women, people with disability, people with severe mental illness, care leavers, or those from deprived or ethnic minority groups including enhanced 1:1 support and financial support. Policy drives to reduce waiting times, promote use of services, identify people using population health management tools, and improve services using user feedback | Local gov, NHS | Ind | High |
| NHS Diabetes Prevention Programme. 9-month lifestyle change programme for people with 'pre-diabetes', delivered face-to-face or digitally, with the aim of preventing diabetes onset | NHS; Local gov | Ind | High |
| NHS Type II DM Path to Remission Programme. 12-week low calorie dietary replacement shakes for obese people with newly diagnosed type 2 diabetes, to achieve weight loss and diabetes remission | NHS | Ind | High |
| **Policies for alcohol and tobacco** |  |  |  |
| Prohibition of the sale of alcohol or tobacco to people aged under 18, with enforcement checks by Local gov | UK gov, Local gov | Pop | Low |
| Duty (additional tax) built into the cost of cigarettes and alcohol products, which typically increases each year in line with the retail price index. Changes in 2024 meant that alcohol duty is tiered according to alcohol strength of the product | UK gov | Pop | Low |
| Prohibitions on advertising and sponsorship from tobacco products, including plain packaging requirements; and advertising restrictions on alcohol products to <18s | UK gov | Pop | Low |
| Legislative responsibility to consult public health teams in Local gov within the licensing process for new and existing venues that sell alcohol, in order to support alcohol harm reduction strategies | UK gov | Pop | Low |
| Prohibitions on smoking in public places (including healthcare settings, in cars where children are present, playgrounds, and government owned premises including workplace vehicles) | UK gov, NHS, Local gov | Pop | Low |
| Specific guidance limiting interactions between policymakers, particularly in the department of health, and representatives of tobacco companies, in order to limit industry motivations influencing policy | UK gov | Pop | Low |
| *NHS Health Checks. Offered by GPs, pharmacies, community health hubs every 5 years to people 40-74 without pre-existing chronic disease. Screen for tobacco and alcohol use; refer to self-help resources and treatment services | NHS | Pop | High |
| Outreach public health support to workplaces to deliver health checks, healthy lifestyle advice, support to establish health champions and workplace health challenges, and signposting (e.g. to smoking cessation support) | Local gov | Pop | High |
| National, regional, and local media campaigns, e.g. 'Better Health', 'Stoptober', 'Protect Little Lungs' campaigns. Adverts, school banner design competitions, signposting to collections of information, advice, risk scores, NHS-approved digital health apps for individuals wanting to reduce alcohol consumption or stop smoking | UK gov, NHS, Local gov | Pop | High |
| NHS standard contract mandates providers to maintain organisational plans for delivering MECC**, and Local gov provide training for local professional groups | NHS, Local gov | Pop | High |
| Mandatory teaching in school about the harms of alcohol and tobacco smoking. Offer more targeted support to those judged to be vulnerable to uptake | UK gov | Pop | High |
| Working with local businesses to increase occupational opportunities, including apprenticeships and pathways to employment, across the board but particularly for those who are (or are at risk of) NEET, and those with learning disability and autism - on the basis that this is a key upstream driver of lifestyle choices such as smoking and alcohol | Local gov | Ind | Low |
| Housing First' - offer stable housing to those with alcohol problems in order to provide a stable environment to boost the likelihood of successful lifestyle change (rather than requiring healthy lifestyle change before housing is available) | UK gov, Local gov | Ind | Low |
| Community-based drug & alcohol services which provide person-centred individual, group, or family behaviour change support to quit smoking and/or reduce alcohol consumption, including provision of smoking cessation aids (including vape kits) and medications, and digital/mobile health interventions. Self- or professional- referral. Sometimes delivered within integrated community 'health hubs'. Sometimes including specific support for young people, care leavers, or pregnant women, including more one-to-one support; and community promotion particularly targeting ethnic minority groups. Sometimes including financial incentives. Policy drives to reduce waiting times, promote use of services, reduce access barriers and inequalities, improve continuity of care, and improve services using user feedback | Local gov | Ind | High |
| Specialised routine physical health checks for those with severe mental health problems | NHS | Ind | High |
| Offer treatment for alcohol-use disorders, including in-patient or community-based detoxification and support services, referral to lifestyle services, and signposting to self-help resources. Specific fund for treatment services for rough sleepers | UK gov, NHS, Local gov | Ind | High |
| All patients admitted to hospital who smoke have smoking status recorded and are offered personalised NHS-funded tobacco treatment services with onward referrals to community services. Specialised pathways for expectant mothers and their partners; and for users of specialist mental health services and learning disability services (including the option to switch to e-cigarettes while in inpatient settings) | NHS | Ind | High |
| **Policies for depression and social isolation** |  |  |  |
| Improve leisure services and green space access to promote self-care and foster good mental wellbeing | UK Gov, Local Gov | Pop | Low |
| National campaigns, such as 'Let's Talk Loneliness', and signposting to self-help support, for example the NHS Every Mind Matters Campaign resources including apps, 5 Ways to Mental Wellbeing guidance, advice on improving sleep quality; and support from national and local charities, including Samaritans and MIND. (N.B. these activities are linked in national documents explicitly to dementia risk reduction - see sources) | UK Gov, NHS, Local gov | Pop | High |
| Government funding support, organisation of, and promotion of community-based organisations offering activities to foster social connections and support mental health, including mobile libraries, community transport schemes, Men's Shed, community arts projects and festivals | Gov, local gov | Pop | High |
| Teaching in school about the importance of mental wellbeing, self-care techniques, resilience training, and how to spot signs of mental ill-health, and the effects of isolation and loneliness and how to seek support if they are concerned; and mental health first aid/awareness training for workplaces | UK gov, local gov | Pop | High |
| Installation work to achieve universal availability of ultra-fast fibre connectivity and 5G to reduce digital inclusion and connect individuals to (virtual) services and to each other | Local gov | Pop | High |
| Prompt mental health support, including pharmaco- and psycho-therapies, available from the NHS for those identified as having depression. Urgent support available via calling the NHS 111 option 2 service | NHS | Ind | Low |
| Community-based healthy lifestyle service (e.g. 'healthy hub') or specific mental health support services. Include individual, group-based, and text-based psychological support services. Self- or professional- referral. Specific support (e.g. mental health coordinators annual checks) for children, care leavers, those recently bereaved, trauma victims, those with long-term conditions, carers, older people, and during life transitions (e.g. childhood to adulthood; moving from home to residential homes). Policy drives to reduce waiting times, have single points of contact, promote use of services, reduce inequalities, and improve services using user feedback | Local gov | Ind | High |
| Specific support for those identified as high risk of loneliness (e.g. older people, frequent attenders, veterans) in primary care or by local government through social prescribing type schemes - working with people to develop tailored plans and connect them to local groups and support services; some similar for children from low income families through the Holidays Activities and Food Programmes; and for people with learning disabilities to engage in leisure activities or voluntary work | Gov, NHS, local gov | Ind | High |
| NHS funded Mental Health Support Teams in schools and colleges to provide early intervention and ongoing help, with trials of digital interventions - pilot scheme currently being fully scaled up | NHS | Ind | High |
| Mental health and wellbeing hubs and NHS Practitioner Health Programme providing healthcare professionals with access to specialist mental health support, providing a safe, confidential non-stigmatising service to turn to when they are struggling and need help | NHS | Ind | High |
| Partnership work to provide support for those with serious mental health problems, learning disabilities and autism to find and retain employment and provide support with housing | NHS, local gov | Ind | High |
| Specific mental health services for military personnel, with support offered before and after active service, mental health professionals present on the front line, and up to 6 months after discharge from the military | UK gov | Ind | High |
| **Policies for Traumatic Brain Injury (also see active travel policies above, marked ^)** | | | |
| National road policies such as speed limits and traffic calming infrastructure in urban/high crash areas, outlawing of drink driving, drug driving, and using mobile phones when driving, driving tests, annual MOT checks, and mandatory seatbelts and helmet wearing (for motorbike users) | UK gov, Local gov | Pop | Low |
| National and local media campaigns such as 'THINK!', 'Life Without Zoe'; free training courses, to encourage safer driving and reduce road crashes; and school teaching on road safety for children | UK gov, local gov | Pop | High |
| **Policies for hearing impairment** |  |  |  |
| Routine newborn hearing checks with referrals to specialist care for those with hearing impairment | NHS | Pop | Low |
| Control of Noise at Work Regulations - statutory guidance for employers to assess risk, reduce noise exposure at source, and providing (and mandating the use of) ear protection where this is insufficient. Regular workplace inspections from local authority environmental health teams to ensure regulations are followed | UK gov, Local gov | Pop | Low |
| Diagnosis of hearing loss, and provision of hearing aids and cochlear implants, including their care and maintenance, freely available from the NHS; with maintenance and checks also available from some local authority-supported community organisations. Treat otitis media in children in line with NICE guidance | NHS, local gov | Ind | Low |
| **Policies for air pollution (also see sustainable travel policies above, marked ^^)** |  |  |  |
| Legislated national targets for key pollutants with requirement for local governments to monitor air quality and declare air quality management areas and enforce plans (e.g. action to reduce traffic build-up) where legal limits are breached. Local government also have the power to enforce clean air zones (low emission zones) for areas of air quality concern meaning high-emitting vehicles pay to drive in that area; and smoke control areas where smoke pollution is a concern, placing more stringent rules on smoke production, including only smokeless fuels may be used for domestic burning. Total ban on the sale of coal and other high polluting fuels for indoor domestic use | UK gov, local gov | Pop | Low |
| Net zero (carbon neutrality) policies within the NHS, local government, and schools through combinations of policies including transition to electric fleets and promoting active travel to work, agile working policies and support, converting to renewable energy sources, retrofitting the estate (e.g. insulation, LED lights), modifying supply chains and medical products (e.g. inhalers), reducing waste, community garden, urban greening, and tree planting initiatives | NHS, Local gov | Pop | Low |
| Subsidisation/funding for some household energy/insulation improvements and transition to renewable heating sources (e.g. heat pumps), collective purchasing schemes for solar panels. Ecological interventions such as tree maintenance and planting. Partnerships with the Energy Saving Trust to provide an app with links to support schemes relevant to local residents | UK gov, local gov | Pop | Low |
| Local governments required to assess air quality impacts of new planning development and local plans, and consider air quality in all local planning and housing decisions; industrial installations and permits are subject to emission limit values | UK gov, local gov | Pop | Low |
| Anti-idling laws enable local governments to issue fixed penalty notices for those unnecessarily idling in their vehicles, for example outside of schools | UK gov, local gov | Pop | Low |
| Improving bus and rail infrastructure, to support transitions towards sustainable travel e.g. from older high-polluting buses to cleaner vehicles, installations of EV charge points - including as a requirement for all new homes with parking spaces, Park & Rides, as well as active travel infrastructure | UK gov, local gov | Pop | Low |
| Changing local government and anchor institutions' (through partnership work) purchasing and other processes to reduce their environmental impact, and 'green economy' programmes to bring local businesses and entrepreneurs together to provide funding and networking support to small low carbon businesses and upskill | Local gov | Pop | Low |
| Free 'air pollution alert system' sending text or email messages to residents when monitors report a moderate or above level of poor air quality, to encourage people (particularly those with health conditions) to reduce exposure | Local gov | Pop | High |
| Communication campaigns e.g. 'Clean Air Day' and carbon literacy training, to raise awareness of, promote innovations in, and encourage more use of sustainable transport options; and individual-level actions to reduce carbon footprints and reduce air pollution | Local gov | Pop | High |
| Provide housing assessments where health and social care professionals or local government officials have concerns about poor indoor air quality | NICE | Ind | Low |
| **Policies for education** |  |  |  |
| Free, mandatory education for children from age 4/5 to 16; mandatory continuation of education, or engagement in an apprenticeship or employment, for those aged 16-19. Statutory responsibility on local government to ensure there are sufficient opportunities available, to monitor and address school performance and attainment gaps, and for schools to create supportive environments that foster learning and encourage attendance (with explicit link to improved attainment); and provision of both mainstream and SEND support, and ensuring equitable access for disabled students | UK gov, local gov | Pop | Low |
| Loans for tuition fees and living costs for the first higher education degree undertaken. Paid back at a variable rate which is proportional to future income. Specific bursaries provided for certain courses | UK gov | Pop | Low |
| Free school meals for all children in the first three years of school, with explicit rationale that this helps learning readiness | UK gov, local gov | Pop | Low |
| Free provision of period products at state-funded schools and further education colleges, with explicit intention to improve learning readiness | UK gov | Pop | Low |
| Free home to school (or appropriate centre for those aged 16-18) transport for those who live beyond walking distance from their nearest school (or one of the nearest three schools if from a low-income family); with more flexibility for those with additional needs e.g. through personal travel budgets | Local gov | Ind | Low |
| Financial support (maximum £40/week) for those from vulnerable groups (e.g. those in care, those from low-income families) to stay in education aged 16-19, as well as additional financial support for specific things like school books, accommodation, and childcare for young parents. Additional financial support for students in higher education with childcare support costs, disabled students, or those experiencing financial hardship | UK gov, local gov | Ind | Low |
| Free school meals for children aged 7-18 for those from low-income families with explicit rationale that this helps learning readiness and for those at older ages addresses a barrier to non-attendance. Free breakfast for all children attending participating schools in the 40% most deprived areas (government subsidise 75% and school the remaining 25%) | UK gov | Ind | Low |
| Statutory requirement for local government to monitor attendance, and take action to address low attendance rates. Schools must make high attendance a central part of its ethos, including incentive schemes rewarding individuals/classes with the highest attendance rates, financial penalties for unauthorised non-attendance, work to understand reasons for non-attendance, and referral to external agencies or safeguarding teams where appropriate. Specific guidance where poor mental health is the cause of poor attendance. Requirement for local government to identify those aged 16-19 who are at risk of, or who have not, secured an appropriate post-16 education/training post, to provide them with advice and support to find one | UK gov, local gov | Ind | High |
| Programmes e.g. 'Uni Connect' bringing together universities, colleges, and local partners to deliver outreach programmes (e.g. awareness raising, myth busting, and mentoring) encouraging young people aged 13-18 in deprived areas to consider higher education; Establishment of a new university in Peterborough, aiming to raise educational aspirations for local people who may not otherwise have considered higher education | Local gov | Ind | High |
| Org=Organisation, NHS=National Health System, Gov = Government, Pop = Population-Level, Ind = Individual-level, GP = General Practitioner, BMI = Body Mass Index, BP = Blood Pressure, MECC = Making Every Contact Count, NEET = Not in Education, Employment, or Training, EV = Electric Vehicle. ^Indicates active travel policies, relevant to several risk factor groups. ^^ indicates sustainable travel policies. | | | |

| Supplementary Table 4 – Proposed dementia primary prevention policies in the UK, grouped by risk factor(s) | | | |
| --- | --- | --- | --- |
| Policy/Intervention | Lead org(s) | Reach | Agency |
| **Policies for obesity, physical inactivity, hypertension, and/or diabetes** |  |  |  |
| Advertising restrictions for products high in fat, salt, and/or sugar being shown on any TV and online between 9pm and 5:30am | UK gov | Pop | Low |
| Restrictions on promotions and sponsorship of unhealthy foods within local control | Local gov | Pop | Low |
| **Policies for alcohol and tobacco** |  |  |  |
| Make low alcohol products more available by relaxing the volume percentage requirement (from 0.05% to 0.5%) and addressing any production cost barriers | UK gov | Pop | Low |
| Displaying the Chief Medical Officer's alcohol guidelines, and age restriction labels, to low alcohol products (this is already done on most standard alcohol products through a voluntary agreement with the industry) | UK gov | Pop | Low |
| Smoke free 2030 ambition. Proposal to increase the minimum legal smoking age by one year every year | UK gov | Pop | Low |
| Restriction of vape flavours, packaging, free samples, and point of sale displays, to make them less attractive/reduce exposure to children (on the basis that vaping may be a gateway to cigarettes) | UK gov | Pop | Low |
| Mandated inclusion of quitting information inside tobacco packs | UK gov | Pop | High |
| Financial incentives for pregnant smokers to quit | UK gov | Ind | High |
| **Policies for depression and social isolation** |  |  |  |
| Early support community mental health hubs specifically for children and young people | UK gov | Ind | High |
| **Policies for Traumatic Brain Injury** | | | |
| Develop a national, cross-sport protocol for managing concussion, make government funding to sporting bodies conditional on increasing spending on athlete protection (including from concussion harm), and introduce training modules for best practice for managing concussive trauma for those working in primary and emergency care | UK gov | Pop | Low |
| Communication campaign to ensure everyone involved in sport, including athletes, coaches, and doctors is aware of best practice | UK gov | Pop | High |
| **Policies for air pollution** |  |  |  |
| Banning of purchases of conventional (petrol/diesel) car and van sales by 2035 | UK gov | Pop | Low |
| Establish community and industrial heating networks, particularly in domestic areas reliant on oil heating systems because they are not connected to the gas network (which could be decarbonised), utilising heat pump networks | Local gov | Ind | Low |
| Replacement schemes offering financial or other incentives to replace older, more polluting cooking stoves with cleaner alternatives; and restrictions on the types of stoves available for sale | UK gov | Ind | Low |
| Org=Organisation, NHS=National Health System, Gov = Government, Pop = Population-Level, Ind = Individual-level | | | |
